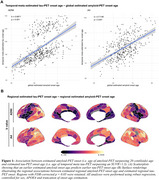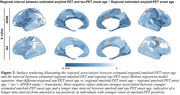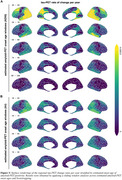# Heterogeneity in tau onset, patterns and accumulation rates are explained by age of amyloid onset

**DOI:** 10.1002/alz70856_104868

**Published:** 2026-01-07

**Authors:** Zeyu Zhu, Amir Dehsarvi, Sebastian Roemer‐Cassiano, Anna Dewenter, Anna Steward, Fabian Hirsch, Lukas Frontzkowski, Davina Biel, Michael Schöll, Günter U Höglinger, Matthias Brendel, Nicolai Franzmeier, for the Alzheimer's Disease Neuroimaging Initiative (ADNI)

**Affiliations:** ^1^ Institute for Stroke and Dementia Research (ISD), University Hospital, LMU Munich, Munich, Bavaria, Germany; ^2^ Department of Neurology, University Hospital, LMU Munich, Munich, Bavaria, Germany; ^3^ Max Planck School of Cognition, Leipzig, Sachsen, Germany; ^4^ Department of Nuclear Medicine, University Hospital, LMU Munich, Munich, Bavaria, Germany; ^5^ University of Gothenburg, Gothenburg, Västra Götalands län, Sweden; ^6^ Dementia Research Centre, Queen Square Institute of Neurology, University College London, London, United Kingdom; ^7^ Department of Psychiatry and Neurochemistry, University of Gothenburg, Mölndal, Västra Götalands län, Sweden; ^8^ Munich Cluster for Systems Neurology (SyNergy), Munich, Bavaria, Germany; ^9^ Institute for Stroke and Dementia Research (ISD), LMU University Hospital, LMU, Munich, Bavaria, Germany

## Abstract

**Background:**

Amyloid pathology drives tau accumulation, i.e., the key driver of clinical worsening in Alzheimer's disease (AD). Yet, there is considerable heterogeneity in the time of tau onset, as well as in the rates and patterns of tau accumulation, which jointly determine symptom onset and clinical trajectories. The exposure to amyloidosis is predictive of AD progression and may therefore predict tau onset and trajectories. Therefore, we investigated how the age and duration of amyloid onset influence tauopathy onset and accumulation.

**Methods:**

We included 479/390 ADNI/A4 participants with Flortaucipir tau‐PET, and Florbetaben/Florbetapir amyloid‐PET. Using sampled iterative local approximation, we determined subject‐specific estimated onset ages of amyloid‐PET positivity (centiloid>20), and tau‐PET positivity (SUVR>1.3). Using robust linear regression, we investigated the associations between estimated amyloid‐PET and tau‐PET onset ages, the delay between amyloid and tau onset and the effect of amyloid onset on tau‐PET change rates.

**Results:**

Younger estimated age of amyloid onset predicted younger estimated age of tau onset in the temporal meta ROI (ADNI/A4, b=0.6871/0.7148, *p* <0.001/0.001, Figure 1A). This result pattern was pronounced in tau vulnerable temporo‐parietal regions, while sparing late Braak regions (Figure 1B). However, a younger estimated age of amyloid onset also predicted a longer delay between amyloid and tau onset, indicating that patients with young onset amyloidosis require longer to develop abnormal tau (Figure 2). By combining sliding window analyses across amyloid onset ages and bootstrapping, we identified that younger amyloid onset is linked to faster tau accumulation, with stronger involvement of parieto‐frontal vs. more pronounced temporal lobe tau accumulation in individuals with later amyloid onset (Figure 3).

**Conclusions:**

Earlier amyloid onset predicts earlier tau onset and faster more neocortically pronounced tau accumulation. At the same time, younger amyloid onset is linked to a longer delay to tauopathy compared to individuals with older‐age amyloid onset. A longer delay between amyloidosis and tauopathy in patients with earlier onset of amyloidosis may widen the window of opportunity for anti‐amyloid drugs to prevent more aggressive tauopathy in these at risk individuals.